# Supplementary material for: Evaluation of Sputtering Processes in Strontium Iridate Thin Films
Source: Nanomaterials (Basel). 2024 Jan 23;14(3):242. doi: 10.3390/nano14030242 (PMC10856326; doi:10.3390/nano14030242)
Supplement: Supplementary file 1 [file nanomaterials-14-00242-s001.zip › nanomaterials-2826254-supplementary.pdf]

Evaluation of sputtering processes in strontium iridate thin films

*Víctor Fuentes 1, Lluis Balcells 1, Zorica Konstantinović 2, Benjamín Martínez 1 and Alberto Pomar 1,**

1 Instituto de Ciencia de Materiales de Barcelona, ICMAB-CSIC, Campus Universitario UAB, 08193 Bellaterra, Spain
2 Center for Solid State Physics and New Materials, Institute of Physics Belgrade, University of Belgrade, Pregrevica 118, 11080 Belgrade, Serbia
* Correspondence: apomar@icmab.es

Supplementary Information

Target Fabrication

A target of nominal composition Sr_2IrO_4 was prepared. We have started from $SrCO_{3(s)}$ as reactive source for strontium (to avoid the use of unstable SrO). SrCO3 was calcined at 650ºC in air atmosphere to reach dehidratation and decomposition into SrO,

$$SrCO_{3(s)} \rightarrow SrO_{(s)} + CO_{2(g)}$$

Then, $SrO_{(s)}$ is mixed stoichiometrically with IrO_2 and heated to high temperatures between 800°C-980°C to achieve the solid chemical reaction,

$$2SrO_{(s)} + IrO_{2(s)} \rightarrow Sr_2IrO_{4(s)}$$

After reacting during every 12h, the powder was again mixed again and the whole thermal treatment was repeated several times to improve phase purity. At each step, the resultant powder was analyzed by X-Ray diffraction. Figure S1 depicts the Θ-2Θ scans of the resultant powder after successive cycles of mixing and heating. In all the spectra the Sr_2IrO_4 phase was the dominant one, being the peak (103) the most intense one (as predicted). In this figure, it can be observed that at the beginning of the process, other Ruddlesden-Popper phases may be identified as impurities. Nevertheless, after several cycles these phases are progressively transformed into the n=1 phase. At the end of the process, the target is mainly Sr_2IrO_4 with an impurity level lower than 5%. Finally, powder was compressed on a hydraulic press to produce a 1-1.3 inch target and it was hardened by a sintering process at 980°C during 48h.

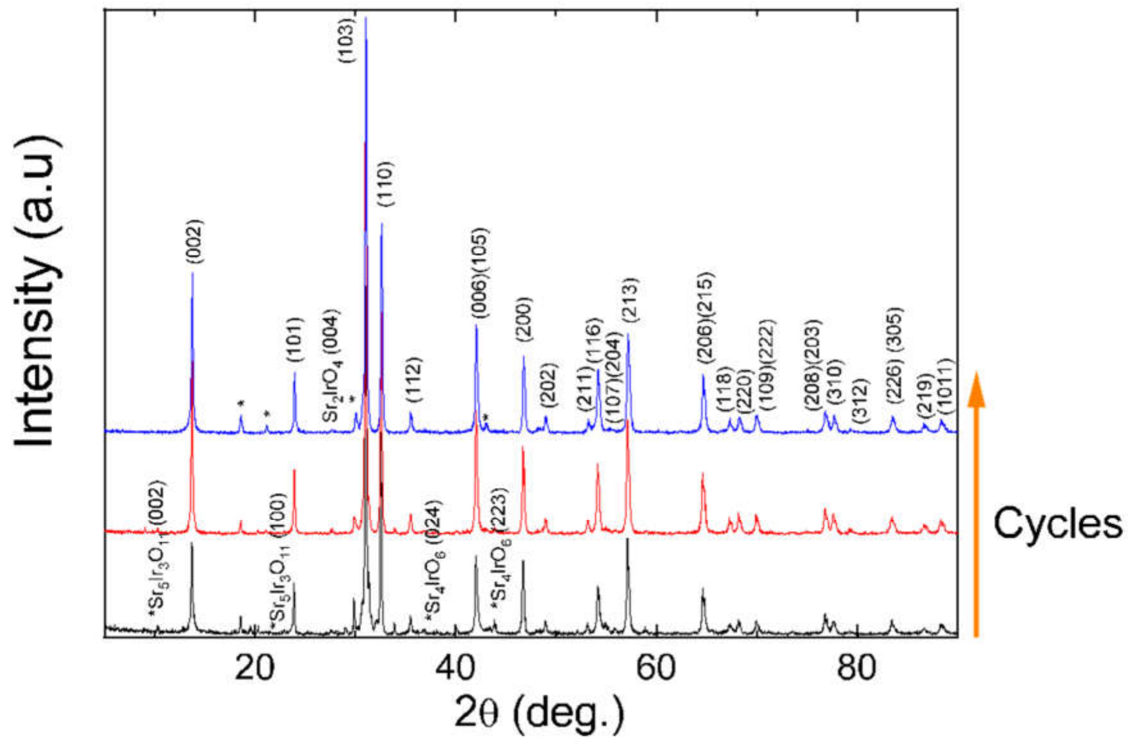

Figure S1: Θ-2Θ scans of the target powder after several mixing and reaction cycles. Peaks are labelled with the corresponding Sr_2IrO_4 reflections (pseudocubic notation).

Influence of oxygen pressure during growth

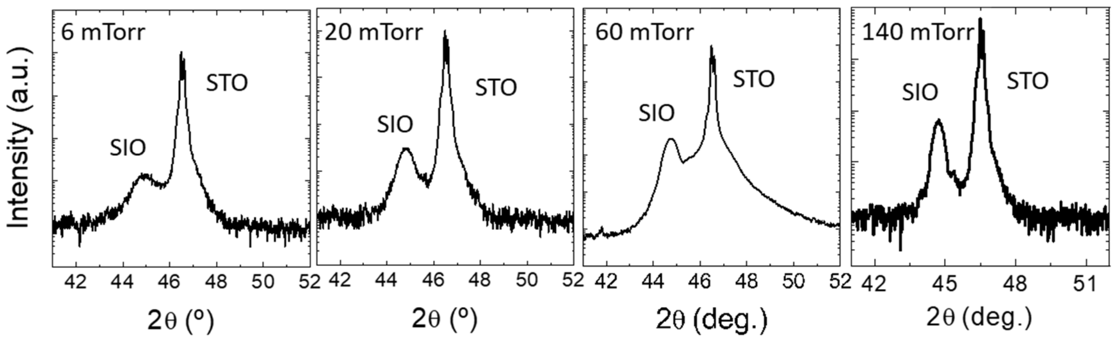

Figure S2: Θ-2Θ scans of a series of SIO-113 thin films deposited at different oxygen pressures and fixed temperature of 900ºC.

The change in background oxygen pressure during growth resulted in a better crystallinity of the SIO-113 films although only peaks associated with the perovskite-like n=∞ phase could be identified.
